# Persistent CD4^+^ lymphopenia is associated with recurrent *Nocardia farcinica* infection and acquired resistance in an AIDS patient: a case report with immunological warning

**DOI:** 10.3389/fimmu.2026.1894622

**Published:** 2026-07-22

**Authors:** Dong Wu, Xiaowu Wang, Tuantuan Li, Xiaojuan Wang

**Affiliations:** 1 Department of Pharmacy, Fuyang People’s Hospital, Fuyang, Anhui, China; 2 Department of Clinical Laboratory, The Second People’s Hospital of Fuyang, Fuyang Infectious Disease Clinical College of Anhui Medical University, Fuyang, Anhui, China; 3 Department of Academic Affairs, Fuyang People’s Hospital, Fuyang, Anhui, China

**Keywords:** acquired drug resistance, aids, CD4+lymphopenia, cerebral toxoplasmosis, immune memory loss, *Nocardia farcinica*

## Abstract

After severe depletion of CD4 T cells in AIDS patients, they are not only prone to a first-time *Nocardia* infection, but also, even if cured, unable to form protective immune memory, leaving them susceptible to reinfection with the same pathogen. More seriously, in the absence of immune surveillance, irregular drug use can accelerate the selection of drug-resistant strains. A 32-year-old man with AIDS and persistent CD4+ count below 100 cells/μL for over three years (nadir 2 cells/μL) developed right lower lobe pneumonia caused by *Nocardia farcinica* four years before the current admission, which was cured with a TMP-SMX-containing regimen. The isolate was sensitive to trimethoprim−sulfamethoxazole (TMP−SMX), and the lesion nearly resolved after treatment. He was prescribed long−term TMP−SMX prophylaxis at discharge but stopped taking it on his own. One year before the current admission, he received sulfadiazine plus pyrimethamine for clinically diagnosed cerebral toxoplasmosis, but his adherence was poor and irregular. On current admission (day 1), he was readmitted with high fever and sepsis. Chest CT showed multiple cavities in the left lower lobe. Blood cultures flagged positive at 25 hours and were identified as *Nocardia farcinica*. The microbiologist reviewed his old records, found the previous nocardial history, and recommended bronchoalveolar lavage (BAL). BAL metagenomic next−generation sequencing again identified *Nocardia farcinica*, but susceptibility testing now showed resistance to TMP−SMX (MIC ≥8/152). He improved after switching to imipenem plus amikacin. He received intravenous imipenem plus amikacin for 14 days, followed by oral linezolid for 6 weeks. At the last follow-up (approximately one year after discharge), his CD4^+^ had risen to only 11 cells/μL, and he had no further nocardial infection. This case shows that when CD4^+^ stays below 100 for a long time, even a first nocardial infection can be cured but may leave insufficient immune memory, rendering the patient susceptible to subsequent infection. The distinction between true reinfection and late relapse could not be definitively established in the absence of strain-level homology data. Irregular, sub−therapeutic sulfonamide exposure, combined with a non−functional immune system, can select for resistant strains.

## 1 Introduction

Nocardiosis is an opportunistic infection caused by *Nocardia* aerobic actinomycetes, which mainly occurs in patients with T cell-mediated immune deficiency (1). CD4+T cells in patients with advanced AIDS are severely depleted, constituting one of the highest risk groups for nocardiosis (2). CD4+T cells play two key roles in anti-infection immunity: eliminating the pathogen in primary infection and forming memory T cells to prevent reinfection (3). When CD4+is continuously lower than 100 μ L, both functions will be severely damaged.

Trimethoprim-sulfamethoxazole (TMP-SMX) is a first-line drug for the treatment and prevention of nocardiosis (4). *Nocardia farcinica* is one of the most virulent and easy to spread strains of *Nocardia*, and it is also the most common *Nocardia* strain in China (5, 6). In recent years, the reports of resistance to sulfonamides have gradually increased, and the emergence of resistance in the process of long-term treatment has been confirmed by experimental evolutionary research (7).

We report a case of an AIDS patient who experienced two successive episodes of *Nocardia farcinica* infection. The focus is to analyze from the perspective of immunology: why does the continuous low CD4+lead to recurrent infection? Why can irregular drug use screen out drug-resistant bacteria?

## 2 Case description

### 2.1 First detection of *Nocardia*

Four years before the current admission, a 32 year old male was admitted to our hospital due to fever, cough, hemoptysis and right chest pain. His CD4+was only 10/μ L, and HIV was newly diagnosed. Chest CT showed a large consolidation in the posterior basal segment of the right lower lobe (approximately 3.3×6.4 cm) with internal necrotic areas, and enlarged right hilar and mediastinal lymph nodes.

The laboratory received his bronchoalveolar lavage fluid. Gram staining of the smear showed branching, beaded positive bacilli, and weak acid-fast staining was also positive. At that time, there was no mass spectrometer in the laboratory, and only “*Nocardia* species” could be reported. The sample was sent to a third-party company for second-generation sequencing, and the result was *Nocardia farcinica* with 1110 sequences. This was the first time the laboratory had detected *Nocardia* from this patient.

Based on this result, the patient received intravenous imipenem plus amikacin for 14 days, followed by oral TMP-SMX alone for another 6 weeks. A CT scan at discharge showed significant reduction of the right lung lesion. Long-term oral TMP-SMX (3 tablets three times daily) was prescribed for secondary prevention along with antiretroviral therapy; however, the patient self-discontinued all medications.

### 2.2 Intermediate period

After discharge, the patient stopped both prophylactic and antiretroviral drugs. Approximately one year before the current admission, his CD4+had dropped to 8/μ L, and he was hospitalized because of fever and cough. A CT scan at that time showed that the old right lung lesion had been largely absorbed, but a new shadow appeared in the left lower lobe. Repeated sputum tests for acid-fast bacilli, fungal (1,3)-β-D-glucan test (G test) and galactomannan test (GM test), and *Nocardia* culture were negative.

Approximately one year before the current admission (a few months after the above hospitalization), he began to have headache and nausea. Contrast-enhanced brain magnetic resonance imaging (MRI) showed multiple ring-enhancing lesions, some with a “target sign”. Serum *toxoplasma* IgG and IgM were both negative. However, given the typical imaging findings and severe immunosuppression (CD4 + 8/μL), a clinical diagnosis of cerebral toxoplasmosis was made, and he was started empirically on sulfadiazine plus pyrimethamine. He took the medications irregularly and often missed doses. Lumbar puncture was offered but refused by the patient.

### 2.3 Second encounter: the clinical laboratory proactively reviewed the medical records

On the current admission (day 1), the patient presented with high fever (41 °C) and productive cough. Chest CT showed a large consolidation in the left lower lobe with multiple small cavities, left hilar and mediastinal lymphadenopathy, and splenomegaly. The original right lower lobe lesion had been absorbed into a small fibrotic cord. Because of the cavities, tuberculosis was suspected clinically. Three sputum acid-fast bacilli smears, Xpert, and IGRA were all negative. The diagnosis was stalled.

**Figure 1:**
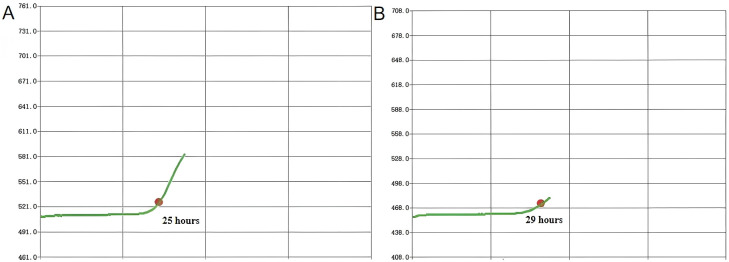
Blood culture growth curves. **(A)** Anaerobic bottle flagged positive at 25 hours. **(B)** Aerobic bottle flagged positive at 29 hours. Both curves show the time to positivity, indicating rapid growth of *Nocardia farcinica*. All calendar dates have been removed.

**Figure 2:**
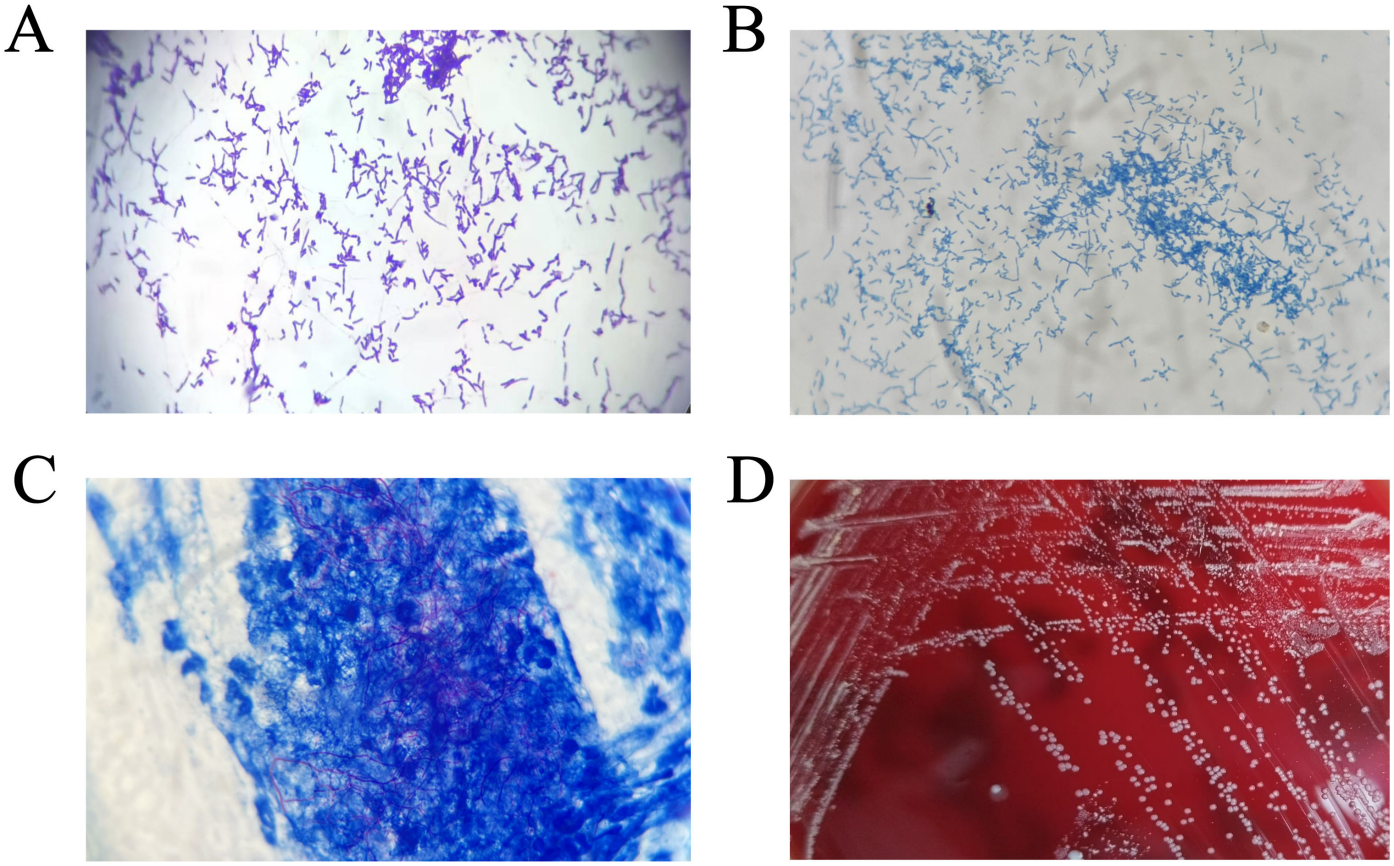
Microbiological identification of *Nocardia farcinica* from the second episode (current admission). **(A)** Gram stain of blood culture isolate: branching, beaded, gram-positive rods (×100). **(B)** Modified Kinyoun weak-acid-fast stain of the same isolate: positive (red filaments); Ziehl-Neelsen stain was negative (not shown). **(C)** Weak-acid-fast stain of bronchoalveolar lavage fluid directly from the patient: branching hyphae (×100). **(D)** Colony morphology on sheep blood agar after 72 hours: dry, chalky, adherent colonies, characteristic of *Nocardia*.

**Figure 3:**
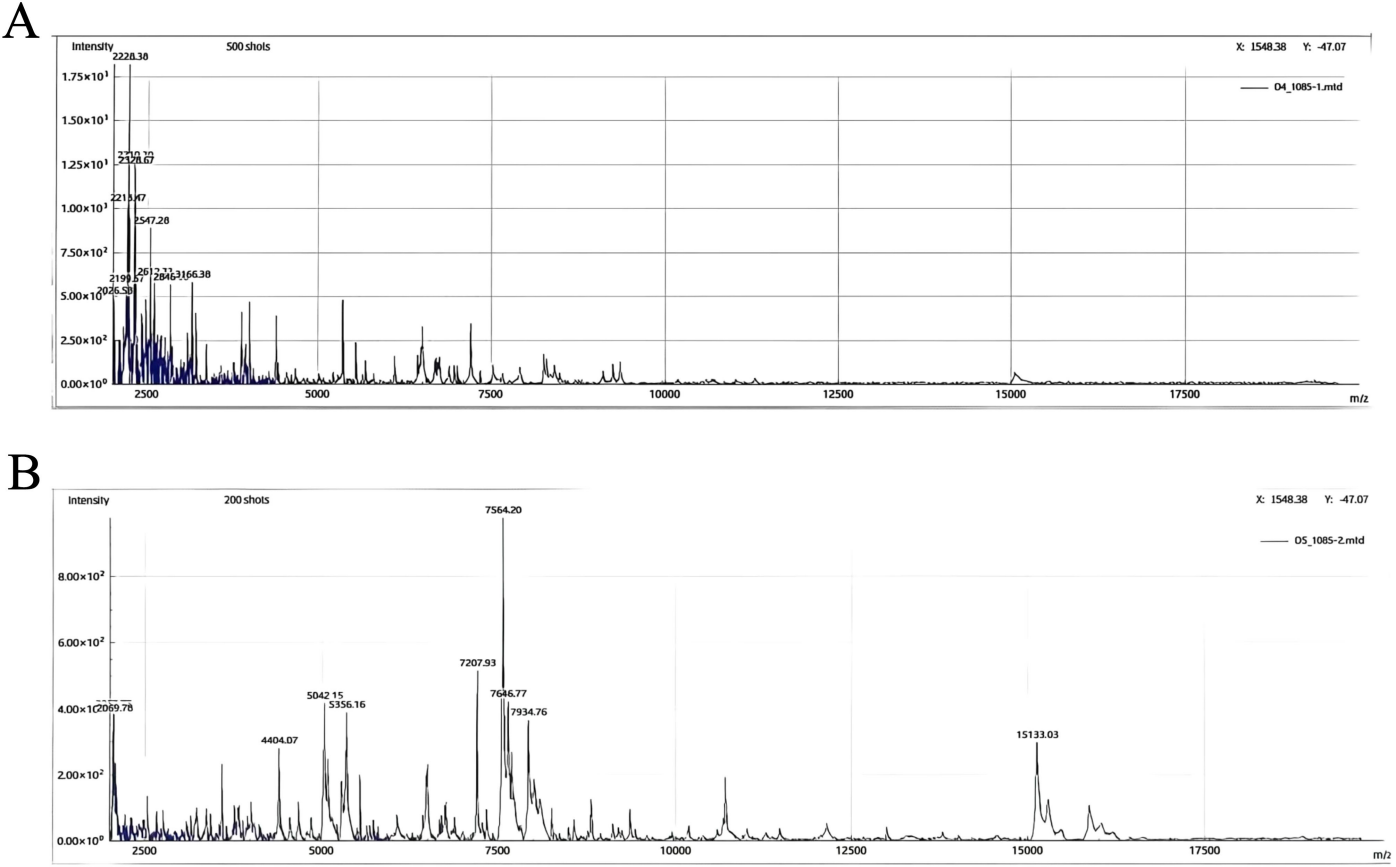
MALDI-TOF mass spectrometry identification of *Nocardia farcinica*. **(A)** Mass spectrum of the blood culture isolate (score 2.09). **(B)** Mass spectrum of the same isolate (duplicate, score 2.07). Both spectra confirm the species as *Nocardia farcinica* with high confidence (score ≥ 2.0).

On day 3 of the current admission, two sets of blood cultures (aerobic+anaerobic) were drawn. By this time, the laboratory had acquired a mass spectrometer. The anaerobic bottle flagged positive at 25 hours and the aerobic bottle at 29 hours (both on day 4) (**Figure 1**). Smear of the positive bottle: Gram staining showed branching, beaded positive bacilli (**Figure 2A**); Ziehl-Neelsen staining was negative, and modified Kinyoun staining was positive (**Figure 2B**). MALDI-TOF MS identified *Nocardia farcinica* (hereafter *Nocardia farcinica*) with scores of 2.09 and 2.07(**Figures 3A, B**). Given the patient’s history of *Nocardia farcinica* pneumonia four years earlier and clinical suspicion of recurrent infection, the patient underwent bronchoscopy with bronchoalveolar lavage (BAL) on day 9.

The lavage fluid was directly smeared with weak acid-fast staining, and branching hyphae were seen (**Figure 2C**). Second-generation sequencing of the lavage fluid (reported on day 11) was again *Nocardia farcinica*, with 2368 sequences and an abundance of 42.14%. Susceptibility testing showed resistance to TMP-SMX (MIC ≥ 8/152); imipenem, amikacin, and linezolid were sensitive. Typical dry, chalky, agar-embedded colonies grew on blood agar after 72 hours (**Figure 2D**). TMP-SMX was discontinued and replaced with intravenous imipenem plus amikacin for 14 days, followed by oral linezolid for 6 weeks at discharge. After 5 days, body temperature normalized and cough significantly improved.

Reinfection was considered the most plausible clinical interpretation based on clinical and radiological evidence: involvement of different lung lobes (right lung in the first episode, left lung in the current episode), complete radiographic resolution of the initial lesion before the new infection, and negative microbiological tests during the interim period. However, because the first isolate was not preserved, strain-level homology could not be confirmed by whole-genome sequencing, and we cannot definitively exclude late relapse of the same strain.

During the current admission, a contrast-enhanced brain MRI was performed on day 3, showing nodular T1 hypointense and T2 mixed hyper/hypointense lesions in the right frontal lobe, right insula, and right cerebellar hemisphere with surrounding edema, but no target sign. The radiology report stated: “Findings suggestive of toxoplasmosis” The patient again refused lumbar puncture.

### 2.4 Short term follow-up

The patient was discharged on day 21 of the current admission. Thereafter, the laboratory received no further *Nocardia* culture or sequencing specimens from him. Through the hospital system and telephone follow-up (every 3 months), we learned that his CD4+ had risen to 11/μL at the last follow-up (approximately one year after discharge), and he had no further nocardial infection through the end of follow-up. However, his adherence remained very poor; he often missed antiretroviral and prophylactic medications, and his CD4+ never exceeded 20/μL. The laboratory added a reminder in the LIS system: nocardiosis may return at any time in this immune state.

The key events, CD4^+^ trajectory, microbiological findings, and antibiotic exposure over the four-year clinical course are summarized in **Table 1**.

**Table 1:** Clinical timeline.

Relative time	Key clinical event	CD4^+^ (cells/μL)	Microbiology/radiology	Antimicrobial therapy/exposure
4 years before current admission	First infection: right lower lobe pneumonia	10	BALF mNGS: N. farcinica (susceptible)	Imipenem + amikacin (14 days) → TMP-SMX (6 weeks)
4 years before (post-treatment)	Lesion resolution, discharge	~10	Chest CT: marked reduction of right lung lesion	TMP-SMX secondary prophylaxis prescribed (patient self-discontinued)
~1 year before current admission	New left lower lobe shadow, fever, cough	8	Sputum cultures, G/GM, *Nocardia* culture: all negative	None
~1 year before (months later)	Headache, nausea; brain MRI: ring-enhancing lesions (clinical diagnosis of cerebral toxoplasmosis)	8	MRI: multiple ring-enhancing lesions, target sign	Sulfadiazine + pyrimethamine (irregular)
Current admission day 1	High fever, productive cough; left lower lobe cavities	10	Chest CT: left lower lobe consolidation with multiple cavities	None
Current admission days 3–4	Blood culture positive	—	Blood culture: *Nocardia farcinica* (MALDI-TOF)	—
Current admission day 9	BAL confirmed recurrent infection	—	BALF mNGS: *Nocardia farcinica* (resistant, MIC ≥8/152)	—
Current admission days 9–23	Antimicrobial therapy	—	—	IV imipenem + amikacin (14 days) → oral linezolid (6 weeks)
Current admission day 21	Discharge	10	—	—
~1 year after discharge (last follow-up)	No further infection	11	—	None (poor adherence)

All exact dates have been removed to protect patient privacy. Normal CD4+ reference: >500 cells/μL. G/GM: (1,3)-β-D-glucan test/galactomannan test. BALF, bronchoalveolar lavage fluid; mNGS, metagenomic next-generation sequencing; MIC, minimum inhibitory concentration.

## 3 Discussion

This case vividly demonstrates two intertwined immunological failures in patients with advanced AIDS: one is the loss of protective immune memory against previously cured intracellular pathogens; Second, in the absence of immune surveillance, irregular antibiotic exposure quickly screened out drug-resistant strains.

The CD4+count of patients continued to be lower than 100/μ L for more than three years, and the lowest was only 2/μ L. This level is clearly listed as a high-risk threshold for initiating multiple opportunistic infection prevention measures in NIH guidelines (4). However, the loss of immune memory cannot be fully explained by a simple reduction in number. In chronic infection, immune checkpoint molecules such as PD-1, CTLA-4 and Tim-3 are highly expressed on the surface of T cells. The up regulation of these “brake” receptors inhibits the proliferation and effector function of T cells, which is the core feature of T cell depletion (8). Even after art treatment and HIV inhibition, the memory T cell function of some patients is still difficult to recover. The research published by Kiessling et al. In nature communications in 2024 directly proved this point: in HIV infected patients receiving art, the recall response of antigen-specific CD4+ T cells was significantly impaired, and transcriptome analysis showed that there was a consistent damage in IFN - α and IFN - γ response pathways; More importantly, *in vitro* treatment with anti-inflammatory drugs can restore the ifn- γ response of memory CD4+T cells, indicating that the inflammatory environment is the key factor driving this defect (9). This means that even if the number of cells has recovered, their ability to recognize and respond to pathogens encountered in the past has been compromised. In addition, even under effective art, HIV-1 specific CD8+ T cells also show tissue resident characteristics and epigenetic scars, suggesting that immune injury is persistent (10).

After the complete imaging absorption of the right lung lesion in four years before current admission, the same *Nocardia* infected the left lung 18 months later (i.e., approximately 2.5 years before current admission). Patients with normal immune function usually have a certain degree of protection after infection with *Nocardia*, but the patient’s immune system behaves as if it had never seen this pathogen. This also confirms that CD4+T cell dependent memory is indispensable for preventing reinfection of the same intracellular pathogen (3).

*Nocardia* is a facultative intracellular pathogen that can survive in macrophages and escape immune clearance. The review of the molecular mechanism of *Nocardia* and host interaction points out that *Nocardia* invades host cells, evades immune defense and survives in host tissues through a series of virulence factors (including mammalian cell entry protein, antioxidant enzymes, phospholipase C, hemolysin and iron carrier related proteins) (1). *Nocardia farcinica* itself is considered to be one of the most pathogenic strains in *Nocardia* genus. Studies have found that nitric oxide plays an important role in the pathogenesis of *Nocardia farcinica* infection, which can aggravate lung pathological damage by inducing nitric oxide production (11). Another study identified nfa34810 as a key virulence factor of *Nocardia farcinica*, which can activate nf- κ B and MAPK pathways through TLR4, promote host cell invasion and stimulate tnf- α secretion (12). These virulence factors endow *Nocardia farcinica* with strong ability of tissue invasion and dissemination, which can explain why the patient’s second infection is not a simple local recurrence, but blood borne dissemination.

A large retrospective cohort reported 15 recurrences (5.0%) among 303 nocardiosis cases, with at least 4 cases involving *Nocardia farcinica* and recurrence times ranging from 18 to 854 days (13). That study found that early recurrences (<60 days) were mostly with the same species (suggesting relapse), whereas late recurrences (2–3 years) were often with a different species (suggesting reinfection). It identified CD4+≤200/μL as a significant predictor of recurrence but did not assess immune memory status or document susceptibility evolution. A propensity-matched analysis found no benefit of secondary prophylaxis (HR 0.96, 95% CI 0.24-3.83). An HIV-associated nocardiosis case series reported 30 patients (mean CD4 + 109/μL), 14 of whom relapsed rapidly (7–38 days) after treatment discontinuation, and most (11/14) failed re-treatment (14). That study highlighted the importance of prolonged therapy but did not address immune memory or resistance evolution. Regarding molecular identification of reinfection, Tamakoshi et al. reported a case of heterologous pulmonary nocardiosis reinfection in an immunocompetent patient with bronchiectasis (*Nocardia cyriacigeorgica → Nocardia beijingensis*), confirmed by 16S rRNA sequencing (15). Both episodes were TMP-SMX susceptible, with no resistance development. This contrasts with our case of homologous recurrent infection - heterologous reinfection suggests different environmental exposure, whereas homologous recurrent infection more directly points to loss of immune memory. Mehta et al. experimentally demonstrated that TMP-SMX resistance can evolve under selection pressure (7). Kiessling et al. showed at the mechanistic level that even with effective ART, HIV-infected individuals have defective antigen-specific CD4+T-cell recall responses (impaired IFN-α/IFN-γ pathways), providing a direct theoretical explanation for why our patient failed to form protective immune memory despite partial CD4+recovery (9).

The novel contribution of our case lies in three aspects. First, regarding mechanistic linkage, we directly connect clinical recurrent infection to the molecular mechanisms of T-cell exhaustion and loss of immune memory. This is supported by evidence that even with effective ART, HIV-infected individuals have defective antigen-specific CD4^+^ T-cell recall responses (impaired IFN-α/IFN-γ pathways) (9). These findings explain why our patient failed to form protective immune memory despite partial CD4^+^ recovery a mechanistic insight that previous recurrence reports, which only described phenomena, did not explore. Second, regarding the clinical evidence chain, using different lobe involvement, complete radiographic resolution of the initial lesion, and negative interim cultures, we constructed a clinically inferable model of immune memory loss in the absence of strain-level homology data, offering a practical inferential approach for similar cases. Third, regarding resistance evolution, we document the full trajectory from sensitive to resistant (TMP-SMX MIC increased from susceptible to ≥8/152) and explain how absent immune surveillance accelerates resistance selection under irregular drug exposure most recurrence reports do not report susceptibility changes or only report resistance at a single time point, rarely documenting the complete sensitive-to-resistant timeline (16).

A fatal combination: immune surveillance deficiency and irregular drug use. The most striking feature of this case is the clear change from the first sensitive strain to the second resistant strain (MIC ≥ 8/152). Since the strains were not preserved in 2021, we cannot confirm whether the same strain acquired resistance or different resistant strains invaded. However, no matter which mechanism, the clinical facts remain the same: the patient has experienced intermittent, sub therapeutic levels of sulfanilamide exposure for three years (first completely discontinuing the prevention, and then taking sulfadiazine+pyrimidine irregularly due to cerebral toxoplasmosis), at the same time, CD4 + is very low, and the immune system is basically ineffective. This directly leads to the special relationship between immunocompromised patients and antibiotic resistance.

Nelson et al’s modeling research published in PLoS One in 2019 specifically discussed this issue. Through mathematical modeling, this study found that immunocompromised patients (taking HIV/AIDS as an example) may contribute more than their proportion to the emergence of antibiotic resistance due to the decline of collective immunity and changes in antibiotic use patterns (16). The reason is that the immune system itself is the “ally” of antibiotics, which can help to remove residual sensitive bacteria when the drug concentration is not enough; Once this ally is missing, the only choice pressure faced by bacteria is the drug itself. Any concentration fluctuation or missed administration will give the opportunity for rapid amplification of drug-resistant mutations. The patient’s medication history perfectly confirms this theory: self-withdrawal after discharge four years before current admission; then irregular sulfadiazine+pyrimethamine intake approximately one year before current admission, resulting in fluctuating blood drug concentrations. This “pulse type” exposure is an efficient way to screen drug resistance mutations. In immunocompetent hosts, even if the drug concentration is insufficient, the residual immune system can clear some sensitive bacteria and delay the emergence of drug resistance; But here, immune surveillance is almost completely absent, and any insufficient antibiotic pressure is directly transformed into selection pressure.

Diagnostic uncertainty regarding the brain lesions. An important limitation of this case is that the clinical diagnosis of cerebral toxoplasmosis made approximately one year before current admission was never microbiologically confirmed. Although the “target sign” on MRI is highly suggestive of toxoplasmosis, serum *toxoplasma* IgG and IgM were both negative. Given that the same patient later developed disseminated *Nocardia farcinica* infection, it is entirely possible that the brain lesions from that time represented nocardial brain abscesses rather than toxoplasmosis. Unfortunately, the patient refused lumbar puncture on two occasions, so no cerebrospinal fluid PCR for *Toxoplasma* or *Nocardia* was available. Therefore, we cannot definitively distinguish between cerebral toxoplasmosis and cerebral nocardiosis in this patient. It is worth noting that if the brain lesions were indeed nocardial in origin, the sulfadiazine+pyrimethamine regimen (both sulfonamide-class drugs) would have had some suppressive effect on *Nocardia*. This could explain why the patient did not develop hematogenous dissemination at that time, and only developed bacteremia and resistance after irregular dosing before the current admission. This further supports our core argument: in immunocompromised hosts, irregular sulfonamide exposure is an important driver of resistance selection.

A limitation of this case is that we cannot definitively distinguish reinfection from late relapse in the absence of strain-level homology data. The clinical evidence involvement of different lung lobes, complete radiographic resolution of the original lesion, and negative interim microbiology is suggestive of reinfection, but it does not exclude the possibility that a persistent nocardial focus (e.g., in macrophages or granulomas) seeded the contralateral lung after a prolonged latency period. *Nocardia* is a facultative intracellular pathogen that can survive in macrophages and escape immune clearance, and some cases of late recurrence have been documented (1, 13). Therefore, we interpret our findings as consistent with recurrent infection, acknowledging that the underlying mechanism true reinfection versus reactivation of a persistent focus cannot be definitively resolved without molecular typing data.

Value of proactive laboratory medicine intervention. The rapid diagnosis in this case was due to the initiative of laboratory staff in reviewing historical medical records after positive blood culture. When the technicians saw branching, beaded Gram-positive bacilli, they did not simply report the result; they discovered the patient’s previous *Nocardia* history in the LIS system, contacted the clinician promptly, redirected the diagnosis from “suspected tuberculosis” to “nocardiosis”, avoided unnecessary anti-tuberculosis treatment, and shortened the time to pathogen identification from days to hours. The concept of diagnostic stewardship is being adopted by more medical institutions. Diagnostic stewardship interventions can significantly shorten the time to pathogen identification and optimize antibiotic use, and proactive chart review is an important component of diagnostic stewardship (17). Moreover, for slow-growing, easily misdiagnosed *Nocardia*, systematic review of previous positive results should become routine for immunocompromised patients.

Clinical implications and limitations. For clinical practice, several points deserve attention: First, when an AIDS patient presents with cavitary pneumonia and tuberculosis screening is negative, nocardiosis should be included in the differential diagnosis as early as possible (1). Second, cure of the first infection does not equal permanent immunity; if similar symptoms recur, etiological investigation must be repeated, including susceptibility testing. Third, for patients with CD4+ persistently below 100/μL, even if antiretroviral therapy is effective, compliance management for secondary prevention should be strengthened to prevent irregular drug use from inducing resistance. For patients who remain severely immunosuppressed despite prescriptions, enhanced measures such as directly observed therapy or simplified regimens may be required. The main limitations of this case include: the first isolate was not preserved, so homology between the two infection episodes could not be confirmed by whole-genome sequencing, and the inference of reinfection is based primarily on imaging and clinical course, with late relapse of the same strain remaining a plausible alternative; the current blood culture and lavage fluid isolates had identical phenotypic and susceptibility profiles, clinically judged as spread of the same strain, but molecular homology was not verified; lack of HIV viral load data precludes assessment of ART efficacy; repeat contrast-enhanced brain MRI was not performed during the current admission to completely exclude central nervous system *nocardiosis* (the patient had no neurological symptoms); compliance data rely mainly on medical records and patient self-report, lacking objective drug concentration monitoring; follow-up time extends only to approximately one year after discharge, so long-term prognosis remains unclear. Despite these limitations, the clinical evidence chain in this case (different lung lobes involved, complete absorption of the original lesion, clear change in susceptibility, and profound persistent CD4+ lymphopenia) provides a vivid clinical illustration for understanding “why immunodeficient patients are recurrently infected with the same pathogen” and “why drug-resistant bacteria are more likely to emerge in the context of immunodeficiency.”

## Data Availability

The original contributions presented in the study are included in the article/supplementary material. Further inquiries can be directed to the corresponding author.
